# Cardiovascular Profile of South African Adults with Low-Level Viremia during Antiretroviral Therapy

**DOI:** 10.3390/jcm11102812

**Published:** 2022-05-16

**Authors:** Shani Botha-Le Roux, Olof Elvstam, Patrick De Boever, Nandu Goswami, Martin Magnusson, Peter M. Nilsson, Hans Strijdom, Per Björkman, Carla M. T. Fourie

**Affiliations:** 1 Hypertension in Africa Research Team (HART), North-West University, Potchefstroom 2520, South Africa; martin.magnusson@med.lu.se (M.M.); carla.fourie@nwu.ac.za (C.M.T.F.); 2 Medical Research Council Unit for Hypertension and Cardiovascular Disease, North-West University, Potchefstroom 2520, South Africa; 3 Department of Translational Medicine, Lund University, 204 02 Malmö, Sweden; olof.elvstam@med.lu.se (O.E.); per.bjorkman@med.lu.se (P.B.); 4 Department of Infectious Diseases, Växjö Central Hospital, 352 34 Växjö, Sweden; 5 Department of Biology, University of Antwerp, 2610 Wilrijk, Belgium; patrick.deboever@uantwerpen.be; 6 Centre for Environmental Sciences, Hasselt University, 3590 Diepenbeek, Belgium; 7 Gravitational Physiology, Aging and Medicine Research Unit, Physiology Division, Medical University of Graz, Neue Stiftingtalstrasse 6, D-5, 8010 Graz, Austria; nandu.goswami@medunigraz.at; 8 Department of Clinical Sciences, Lund University, 205 02 Malmö, Sweden; peter.nilsson@med.lu.se; 9 Department of Cardiology, Skåne University Hospital, 205 02 Malmö, Sweden; 10 Wallenberg Center for Molecular Medicine, Lund University, 221 84 Lund, Sweden; 11 Centre for Cardio-Metabolic Research in Africa, Division of Medical Physiology, Faculty of Medicine and Health Sciences, Stellenbosch University, Cape Town 8000, South Africa; jgstr@sun.ac.za; 12 Department of Infectious Diseases, Skåne University Hospital, 205 02 Malmö, Sweden

**Keywords:** biomarkers, blood vessels, cardiovascular disease, human immunodeficiency virus, viral load

## Abstract

Chronic inflammation is an HIV infection feature, contributing to elevated risk of cardiovascular disease among people with HIV, which can be induced by viral replication. A proportion of antiretroviral therapy (ART) recipients fail to achieve viral suppression, despite not meeting criteria for treatment failure, so-called low-level viremia (LLV). We investigated the relationship between LLV and an array of cardiovascular measures and biomarkers. South Africans with LLV (viral load = 50–999 copies/mL) and virological suppression (viral load <50 copies/mL) were selected from the EndoAfrica study (all receiving efavirenz-based ART) for cross-sectional comparison of vascular structure and function measures, as well as 21 plasma biomarkers related to cardiovascular risk and inflammation. Associations were investigated with univariate, multivariate, and binomial logistic regression analyses (having outcome measures above (cases) or below (controls) the 75th percentile). Among 208 participants, 95 (46%) had LLV, and 113 (54%) had viral suppression. The median age was 44 years, 73% were women, and the median ART duration was 4.5 years. Cardiovascular measures and biomarker levels were similar between these two categories. Cardiovascular function and structure measures were not associated with viremia status and having LLV did not increase the odds of having outcome measures above the 75th percentile. In this study among South African ART recipients, LLV did not associate with cardiovascular risk.

## 1. Introduction

Antiretroviral therapy (ART) has radically improved the health and survival of people with HIV (PWH). With a declining incidence of opportunistic infections and increasing longevity, cardiovascular diseases (CVD) have emerged as an important cause of mortality in PWH [1]. A meta-analysis, including 80 studies, estimated that PWH have at least a twofold higher risk of developing CVD than HIV-negative individuals. However, this meta-analysis included only one study from a lower-middle-income country [2]. Black South Africans face multimorbidity with non-communicable diseases such as HIV and tuberculosis (requiring daily access to medication), as well as communicable diseases such as hypertension and diabetes, which are among the highest causes of mortality [3]. In 2021, South Africa was home to 8.2 million PWH and had the most extensive ART program globally [4]. Several mechanisms for the increased CVD risk among PWH can be considered, including traditional risk factors such as smoking, alcohol consumption, hypertension, and dyslipidemia [5]. However, HIV-specific factors may also involve endothelial dysfunction [6], immune activation [7], chronic inflammation [8], CD4 cell depletion [6], and cumulative exposure to ART [9]. 

Chronic inflammation persists in PWH receiving ART, as serum immune activation markers remain elevated compared to HIV-negative controls even after six years of effective ART [10]. ART leads to undetectable HIV RNA levels in plasma in most PWH. However, a subset of ART recipients fails to achieve viral suppression despite not meeting the criteria for virological treatment failure. This phenomenon is commonly called low-level viremia (LLV). Persons with LLV have an increased risk of virological treatment failure [11]. In addition, some studies have associated LLV with adverse clinical outcomes. In a Swedish HIV cohort, LLV (defined as 50–999 copies/mL) was associated with increased mortality and LLV (defined as 200–999 copies/mL) was associated with severe non-AIDS events, among which 52% were CVD [12]. Furthermore, viral load (VL) > 400 copies/mL has been associated with incident CVD in a Dutch study [13]. 

The underlying mechanisms for linking LLV during ART and CVD are unclear. In some studies from Europe and North America, elevated biomarker levels reflecting immune activation, cardiovascular risk, and coagulation have been observed in persons with LLV compared with virologic suppression [14,15].

It is unknown whether such associations exist in sub-Saharan Africa, the world region where most PWH live. To investigate whether LLV is associated with the cardiovascular risk profile in this population, we aimed to study an array of cardiovascular measurements and biomarkers in South African PWH receiving ART.

## 2. Materials and Methods

A cohort of PWH from the Potchefstroom area in the North West Province in South Africa, who were 18–60 years old and of African descent, were included in this cross-sectional study, which is part of the larger EndoAfrica Study [16]. The detailed EndoAfrica-NWU study protocol has been published previously [17]. For the current study, we excluded participants who were HIV-uninfected (*n* = 104), without VL data (*n* = 13), those not receiving ART (*n* = 9), and participants with high-level viremia (VL > 1000 copies/mL; *n* = 48), leaving a total of 208 participants for inclusion. The World Health Organization guidelines, widely used in Sub-Saharan Africa, currently suggest a threshold of 1000 copies/mL for this definition. We classified viremia groups as LLV (VL = 50–999 copies/mL) and suppressed viremia (VL < 50 copies/mL). All participants were on first-line fixed-dose combination ART (emtricitabine, tenofovir-disoproxil and efavirenz) for ≥4 weeks on the day of the data collection. All participants were in a fasting state during data collection.

This study received permissions from the Health Research Ethics Committee of the North-West University, the North West Department of Health, and the local hospital’s Potchefstroom Patient Group. All participants gave written informed consent to inclusion and data collection from clinic files before enrolment. All sample and data collections took place at the Hypertension Research and Training Clinic on the Potchefstroom Campus of the North-West University.

### 2.1. Demographic and Anthropometric Data Collection

A general health questionnaire collected data on age, sex, tobacco use, alcohol consumption, physical activity level, medication use, and HIV-specific information (duration of HIV infection, ART use, and adherence). The questionnaire information was followed up and confirmed by a research assistant at the participants’ medical clinics.

Standardized procedures were used to measure body weight (SECA 813 Electronic scale, SECA, Hamburg, Germany), height (SECA 213 Stadiometer, SECA, Hamburg, Germany), and waist circumference (Lufkin steel anthropometric tape, W606PM; Lufkin, Apex, NC, USA).

### 2.2. Biochemical Analyses

A detailed description of the methods and technologies used to analyze biomarkers is in the EndoAfrica-NWU study protocol [17]. HIV was diagnosed with rapid tests and confirmed following national guidelines. Newly diagnosed participants were referred to a local medical clinic. Blood samples were prepared at the on-site laboratory of the NWU. Blood samples for VL and CD4 cell count analyses were sent to the National Health Laboratory Service. Plasma aliquots were stored at −80 °C. The following biomarkers were quantified: intercellular adhesion molecule-1, vascular cell adhesion molecule-1, P-selectin, myeloperoxidase, growth differentiation factor-15, and a disintegrin and metalloproteinase with a thrombospondin type 1 motif, member 13 (ADAMTS13), reactive oxygen species, Troponin T, N-terminal proB-type natriuretic peptide (NT-proBNP), interleukin-6, C-reactive protein, total cholesterol, triglycerides, low-density lipoproteins, high-density lipoproteins, apolipoprotein A and B, gamma-glutamyl transferase and creatinine. Glycated hemoglobin was analyzed from hemolyzed (ethylenediaminetetraacetic acid) whole blood and glucose from plasma. We estimated the glomerular filtration rate with the Chronic Kidney Disease Epidemiology Collaboration equation without the race factor.

### 2.3. Cardiovascular Measurements

Brachial systolic and diastolic blood pressure and heart rate were measured in duplicate on the left arm sitting with an OMRON M6 automatic digital blood pressure monitor (Omron Healthcare, Kyoto, Japan). We calculated the mean arterial pressure.

With the participant in a supine position, pulse wave analysis measured central systolic blood pressure and pulse pressure from arterial waveforms using a SphygmoCor^®^ XCEL device (AtCor Medical, Sydney, Australia). Aortic pulse wave velocity was measured from the simultaneous arterial waveforms in duplicate after fitting a femoral cuff and measuring the pulse wave travel distance (80% of the carotid pulse-to-cuff and femoral pulse-to-cuff distances). Measurements were repeated if pressure measures differed by >3 mmHg or pulse wave velocity differed by >0.5 m/s. We calculated femoral-brachial pulse pressure amplification from pulse wave analysis measurements as brachial pulse pressure divided by central pulse pressure.

Sonographic images and cineloops were obtained from the left and right carotid arteries with a General Electric Vivid E9 ultrasound apparatus (GE Vingmed Ultrasound A/S, Horten, Norway). One investigator analyzed the data with the carotid vessel analyzer automated software (Vascular Research Tools 6, Medical imaging applications, Coralville, IA, USA). Carotid intima-media thickness was quantified as the average between the minimum and maximum intima-media thickness measures of the far wall using the mean between the left and right carotid arteries. Simultaneously, the diameter distensibility percentage was determined using the difference between the maximum and minimum diameters, divided by the minimum diameter, and multiplied by 100.

### 2.4. Comorbidities

Hypertension was classified as systolic blood pressure ≥140 mmHg and/or diastolic blood pressure ≥90 mmHg, as prescribed by the 2020 International Society of Hypertension Global Hypertension Practice Guidelines, and/or use of anti-hypertensive medication. We classified central obesity as having a waist circumference of ≥80 cm (women) or ≥94 cm (men) using the South African dyslipidemia guideline 2018 consensus statement. As per the harmonized metabolic syndrome definition, we classified the metabolic syndrome as meeting at least three of the following criteria: waist circumference ≥80 cm (women) or ≥94 cm (men); triglycerides ≥1.7 mmol/L or using statins; high-density lipoprotein <1.3 mmol/L (women) or <1.0 mmol/L (men); systolic blood pressure ≥130 mmHg and/or diastolic blood pressure ≥85 mmHg and/or using anti-hypertensive medication; fasting glucose ≥5.6 mmol/L. As per the American Diabetes Association prescription, participants were identified as diabetic with a glycated hemoglobin of ≥6.5% (48 mmol/mol) and/or receiving anti-diabetic medication.

### 2.5. Statistical Analyses

Data analysis was executed in IBM^®^ SPSS^®^ Statistics software for Windows version 26 (Armonk, NY, USA: IBM Corp.). Data were inspected for normality with QQ plots and skewness, kurtosis, and Shapiro–Wilk test statistics. A *p* < 0.05 showed statistical significance.

Characteristics and cardiovascular-related measures and biomarkers between viremia groups were compared using Mann–Whitney U tests for continuous data and Pearson’s χ^2^ tests for categorical data. In sensitivity analyses, we also examined differences disaggregated by sex within the respective viremia groups. Continuous data are given as medians with 25th and 75th percentile ranges and categorical data as proportions. We performed the Benjamini–Hochberg procedure to account for the effect of multiple testing and decrease the false discovery rate (set at <0.05).

We performed Spearman rank correlation analyses to assess relationships between cardiovascular measures and biomarkers in participants with LLV and univariate and multivariate linear regression analyses explored relationships between cardiovascular measures and biomarkers (central systolic blood pressure, pulse wave velocity, pulse pressure amplification, intima-media thickness, distensibility, Troponin T and NT-proBNP) and viremia status (where 0 = suppressed viremia and 1 = LLV). Non-Gaussian variables (Troponin T, NT-proBNP, and gamma-glutamyl transferase) were logarithmically transformed, and all continuous variables were standardized before entry into the models. Confounding variables included in multivariate analyses were chosen based on the strength of their relationship with the primary outcome variables, as determined by Spearman correlation analyses. Variables in the models included viremia status, sex, age, waist circumference, cholesterol, glucose, gamma-glutamyl transferase, tobacco use, glomerular filtration rate, and mean arterial pressure (for Troponin T, NT-proBNP, pulse wave velocity, intima-media thickness, and distensibility) or mean arterial pressure, heart rate, and height (for pulse pressure amplification). Variance Inflation Factor values were ≤2.24 for all variables in all models, showing low multicollinearity.

In a sensitivity analysis, the above multiple regression analyses were repeated. We excluded participants with C-reactive protein levels of >10.0 mg/L (*n* = 32), because C-reactive protein levels above that threshold could reflect non-specific elevation rather than cardiovascular risk [18]. In another sensitivity analysis, we repeated the multiple regression analysis, but this time excluded menopausal women (>50 years old, *n* = 32). In both cases, the associations with low-level viremia remained insignificant.

Further, standardized binomial logistic regression analyses were performed to determine odds ratios. All cardiovascular measures and biomarkers were dichotomized as ≥75th percentile = 1 and <75th percentile = 0 and then used as outcome measures, while viremia status was entered as the primary predictor with the other predictors in the earlier models.

Using the G*Power 3.1 statistical analysis software, this study was estimated to detect an effect size (f^2^) of 0.11 with a power of 90% using 208 participants as the sample size and alpha error set at 0.05 for multiple linear regression with a maximum of 12 covariates and cardiovascular measures as the primary outcome variables.

## 3. Results

### 3.1. Participant Characteristics

Among the 208 included participants, the median age was 44 (interquartile range = 14) years, with a majority being women (74%), 63% diagnosed with HIV >5 years previously, and 46% received ART for >5 years. The median treatment duration was 4.3 (1.8;7.7) years. Hypertension was diagnosed in 38% of participants (although only 19% reported anti-hypertensive treatment), 53% were centrally obese, and 26% fulfilled the metabolic syndrome criteria. In total, 95 (46%) had LLV with a median VL of 60.0 (50.0;154.0) copies/mL.

### 3.2. Cardiovascular Profile in Participants Regarding Viremia Classification

There were no differences in characteristics between the viremia groups, except for higher alcohol consumption (*p* = 0.012) and fasting glucose (*p* = 0.035) in persons with suppressed viremia compared to those with LLV (Table 1).

The cardiovascular profile of participants with LLV was similar to that of persons with viral suppression, with no significant differences detected for cardiovascular measures (blood pressure, pulse wave velocity, pulse pressure amplification, intima-media thickness, carotid diameter distensibility, Troponin-T, and NT-proBNP), or other cardiovascular-related biomarker levels (C-reactive protein, interleukin-6, intercellular adhesion molecule-1, vascular cell adhesion molecule-1, P-selectin, myeloperoxidase, growth differentiation factor-15, ADAMTS13, and reactive oxygen species).

Cardiovascular function and structure measures were not associated with viremia status (Table 2). We investigated whether LLV increased the odds of cardiovascular risk, i.e., having cardiovascular measures or biomarker levels above the 75th percentile, but found no significant effects (Table 3).

To explore the potential effect of sex, we compared cardiovascular measures and biomarkers between viremia groups in men and women, respectively, with no significant differences observed (Supplementary Table S1).

## 4. Discussion

In this cross-sectional study of South African PWH receiving ART, we observed no differences in a broad range of cardiovascular structure and function measurements and plasma biomarkers reflecting cardiovascular risk or systemic inflammation in persons with LLV compared to those with viral suppression. This study is the first to explore the association between viremia status and cardiovascular risk predictors in South Africa.

Our findings contrast with studies from high-income settings (all with a predominance of male participants), which have reported higher immune activation, inflammation, or other cardiovascular risk biomarkers in persons with LLV. In two studies, elevated interleukin-6 levels were found [19,20], whereas another study observed higher levels of growth differentiation factor-15 [15]. Increased CD8 T-cell activation in persons with LLV has also been reported [21], and in another study, both treated and treatment-naive LLV patients have had persistent platelet activation [22], also contributing to higher cardiovascular risk. Several factors might explain the discordant findings between studies exploring the potential interaction between LLV and cardiovascular risk. In our cohort, participants were still relatively young, most participants were female, and all were of African descent, while some were obese and hypertensive, received anti-hypertensive treatment, and fulfilled the metabolic syndrome criteria. These factors could therefore represent a different cardiovascular risk profile [23].

Methods for assessing subclinical atherosclerosis and vascular function are helpful to assess the risk of incident CVD. For instance, elevated blood pressure and pulse pressure, pulse wave velocity, and carotid intima-media thickness are physiological measurements that can predict future CVD [24]. Studies from high-income settings have shown that PWH have less favorable carotid intima-media thickness, shear strain, presence of plaque, and pulse wave velocity parameters [25,26]. Higher pulse wave velocity and central pulse pressure measures are present in untreated PWH compared to HIV-negative adults [27]. Conversely, one South African study reported no differences in pulse wave velocity and carotid intima-media thickness between PWH and uninfected controls. However, cell adhesion molecule levels were higher in PWH, indicating endothelial activation [28]. Apart from physiological measurements, various biomarkers have been assessed for their capacity to predict CVD. Interleukin-6 and C-reactive protein (both markers of systemic inflammation) have been associated with incident CVD in PWH [8,29].

Several investigations suggest that the degree of viral suppression during ART influences CVD risk among PWH. A positive relationship between carotid intima-media thickness and HIV VL was reported in PWH on ART for ≥12 months, of whom 80% had VL < 50 copies/mL [26]. Higher progression in carotid intima-media thickness was present in individuals with persistent non-suppressed viremia (>400 copies/mL) in a United States study [30]. Furthermore, persons with residual viremia (detectable VL < 20 copies/mL) had higher carotid intima-media thickness than those with undetectable VL in a French cross-sectional study [31]. However, in Brazilian PWH, higher cumulative exposure to viremia, measured as viremia-copy-years, was not associated with carotid intima-media thickness after adjustment for traditional risk factors such as smoking, body mass index, family history of CVD, and hypertension [32].

The factors that determine CVD risk among PWH residing in sub-Saharan Africa are not fully understood. A recent meta-analysis suggested that PWH are twice as likely to develop CVD than HIV-negative individuals, with the highest impact being on people in the sub-Sahara African region [2]. However, a 10-year follow-up of the South African leg of the Prospective Urban and Rural Epidemiology study found no differences between PWH receiving ART and uninfected individuals regarding cardiovascular profile. Differences were noted in lipid and inflammatory markers, with elevated total cholesterol levels, triglycerides, and high-density lipoprotein restricted to PWH, and longitudinal increases in C-reactive protein levels [33]. These observations suggest that the increased CVD risk among PWH observed in Europe and the USA may not be universal. Furthermore, all individuals in our study received efavirenz-based ART. In contrast, cohorts in high-income countries also contain recipients of different ART regimens (including protease inhibitors and abacavir), which could influence CVD risk [34]. Regardless, in this context, persons with LLV may require special consideration. LLV can reflect a greater HIV reservoir, and advanced disease at ART initiation [35]. Furthermore, increased CVD risk is also suggested by studies linking LLV to increased overall mortality and non-AIDS severe events [12,13], but this potential association has not been extensively investigated.

The African Cohort Study recently reported a higher prevalence of elevated blood pressure, hypercholesterolemia, hyperglycemia, and renal insufficiency among people with persistent LLV (VL < 1000 copies/mL) compared to virally suppressed individuals [36]. We did not observe such associations in our cohort. In the African Cohort Study cohort (with most participants from Kenya and Uganda), the proportion of smokers was considerably lower compared to our cohort (4.1% vs. 58%). In both cohorts, relatively high proportions of participants reported treatment interruptions (17% vs. 15%). Alcohol consumption and diabetes are other factors that influence CVD incidence. Alcohol consumption could promote microbial translocation and inflammation synergistically with HIV [37]. In our study, participants with LLV were less likely to report alcohol consumption (54% vs. 72%), were less often categorized as diabetic (3.2% vs. 8.9%) and had lower median glucose levels than those with SV. We did however account for these confounders in our regression models.

LLV can occur via at least two mechanisms that are not mutually exclusive: clonal outgrowth from latently infected cells and ongoing viral replication. Interestingly, these two types of LLV have been linked to different cytokine profiles, suggesting that the association between LLV and systemic inflammation could vary regarding the mechanisms involved [38]. In our study, the proportion of participants reporting treatment interruptions was relatively high, suggesting viral replication enabled by suboptimal plasma concentrations of antiretroviral drugs to be the primary mechanism of LLV in our population.

Some limitations and strengths of this study merit discussion. Firstly, viremia classification was based on a single VL instead of repeated measurements. Some participants classified as having LLV may have manifested transient episodes of viremia, which could be a different entity from persistent LLV. Access to longitudinal VL data would have led to stricter viremia classification. As this is a cross-sectional study, causality cannot be proven, a problem common to all cross-sectional studies. On the other hand, a strength of the study is that this study includes novel data on LLV and an extensive cardiovascular and biomarker profile from the country with the highest global HIV rate. Further, measurements were performed under controlled conditions, by trained and experienced researchers, and with gold standard apparatus and techniques.

## 5. Conclusions

In conclusion, in this cross-sectional analysis of South African adults with HIV, we found no associations between LLV during ART and cardiovascular phenotype, based on a wide range of measurements that reflect cardiovascular structure, function, and CVD risk.

## Figures and Tables

**Table 1:** Difference in characteristics and cardiovascular measures between people living with HIV and receiving ART, stratified by viremia status.

	Suppressed Viremia *n* = 113	Low-Level Viremia *n* = 95	*p*-Value
Men, *n* (%)	32/113 (28.3)	23/95 (24.2)	0.50
Age (years)	45.0 (36.0;52.0)	43.0 (36.0;49.0)	0.33
Waist circumference (cm)	85.1 (77.1;94.2)	82.9 (73.3;93.6)	0.21
Body mass index (kg/m^2^)	25.9 (20.9;31.1)	24.4 (21.2;29.6)	0.99
Body mass index categories, *n* (%)			0.71
*<25.0 kg/m^2^*	53 (46.9)	50 (52.6)	
*25.0–29.9 kg/m^2^*	30 (26.5)	23 (24.2)	
*≥30.0 kg/m^2^*	30 (26.5)	22 (23.2)	
Waist-to-height ratio	0.53 (0.47;0.60)	0.52 (0.45;0.59)	0.36
**HIV profile**			
Viral load (copies/mL)	-	60.0 (50.0;154.0)	**<0.001**
CD4 count (cells/µL)	585 (386;790)	530 (372;712)	0.13
Duration of HIV >5 years, *n* (%)	67/113 (59.3)	63/94 (67.0)	0.25
Antiretroviral therapy, *n* (%)			0.95
*Currently*	96/113 (85.0)	81/95 (85.3)	
*Defaulted*	17/113 (15.0)	14/95 (14.7)	
Duration of ART (years)	4.06 (1.52;7.45)	5.04 (1.98;8.26)	0.32
Duration of ART >5 years, *n* (%)	47/112 (42.0)	47/94 (50.0)	0.25
**Cardiovascular measures**			
Brachial systolic blood pressure (mmHg)	116 (106;130)	114 (105;128)	0.39
Brachial diastolic blood pressure (mmHg)	81 (74;89)	82 (74;92)	0.39
Brachial mean arterial pressure (mmHg)	92 (85;104)	93 (84;102)	0.84
Central systolic blood pressure (mmHg)	113 (107;131)	113 (104;128)	0.57
Central pulse pressure (mmHg)	34 (29;41)	33 (28;39)	0.24
Heart rate (beats/min)	59 (60;79)	71 (64;79)	0.37
Pulse wave velocity (m/s)	7.70 (6.90;8.70)	7.65 (6.43;8.50)	0.54
Pulse pressure amplification	1.34 (1.28;1.44)	1.33 (1.26;1.43)	0.64
Carotid intima-media thickness (mm)	0.60 (0.57;0.65)	0.60 (0.57;0.65)	0.99
Carotid diameter distensibility (%)	9.39 (6.89;12.3)	8.98 (6.45;12.4)	0.35
Troponin-T, >3.00 (pg/mL) ^‡^	4.89 (3.63;6.67)	4.57 (3.78;6.64)	0.95
NT-proBNP (pg/mL)	52.1 (21.6;113.0)	43.3 (19.2;78.7)	0.17
**Other biomarkers**			
C-reactive protein (mg/L)	2.49 (1.25;7.51)	3.17 (0.84;6.77)	0.73
Interleukin-6 (pg/mL)	2.50 (1.60;4.28)	2.74 (1.84;4.80)	0.30
Intercellular adhesion molecule-1 (ng/mL)	29.6 (19.2;52.1)	29.3 (22.4;45.3)	0.79
Vascular cell adhesion molecule-1 (ng/mL)	504 (374;619)	498 (383;638)	0.83
P-selectin (ng/mL)	27.7 (21.5;36.2)	29.7 (20.7;37.1)	0.71
Myeloperoxidase (ng/mL)	68.1 (43.7;97.7)	62.6 (38.0;91.0)	0.17
Growth differentiation factor-15 (ng/mL)	0.65 (0.43;1.05)	0.67 (0.48;0.85)	0.10
ADAMTS13 (ng/mL)	400 (329;501)	399 (359;470)	0.62
Reactive oxygen species (Units)	205 (159;244)	218 (173;254)	0.12
Cholesterol (mmol/L)	2.82 (2.38;3.66)	2.93 (2.53;3.36)	0.45
Triglycerides (mmol/L)	0.75 (0.55;1.10)	0.71 (0.51;1.13)	0.50
Low-density lipoproteins (mmol/L)	1.74 (1.24;2.33)	1.69 (1.44;2.10)	0.49
High-density lipoproteins (mmol/L)	1.03 (0.79;1.30)	1.02 (0.75;1.33)	0.72
Apolipoprotein A (g/L)	1.17 (0.95;1.45)	1.15 (0.94;1.41)	0.68
Apolipoprotein B (g/L)	0.59 (0.45;0.75)	0.60 (0.49;0.75)	0.46
Glycated hemoglobin (%)	5.42 (5.11;5.74)	5.36 (5.18;5.66)	0.53
Glucose (mmol/L)	3.66 (3.26;4.28)	3.44 (3.16;3.94)	**0.035**
Gamma-glutamyl transferase (U/L)	42.4 (26.4;89.9)	37.2 (20.1;120.0)	0.54
eGFR (mL/min/1.73 m^2^)	118 (108;130)	120 (109;130)	0.83
**Comorbidity classifications**			
Hypertensive, *n* (%)	43/113 (38.1)	35/95 (36.8)	0.86
Centrally obese, *n* (%)	62/113 (54.9)	49/95 (51.6)	0.64
Diabetic, *n* (%)	10/112 (8.90)	3/95 (3.20)	0.088
Metabolic syndrome, *n* (%)	29/111 (26.1)	25/94 (26.6)	0.94
**Lifestyle measures**			
Tobacco use, *n* (%)	65/113 (57.5)	54/94 (57.4)	0.99
Alcohol consumption, *n* (%)	81/113 (71.7)	51/93 (54.8)	**0.012**
Physically active, *n* (%)	97/113 (85.8)	76/93 (81.7)	0.42
**Other medication use**			
Anti-hypertensive, *n* (%)	23/113 (20.4)	17/95 (17.9)	0.65
Statins, *n* (%)	3/113 (2.70)	2/95 (2.10)	0.80
Anti-diabetic, *n* (%)	1/113 (0.90)	0/95 (0.00)	0.36
Anti-inflammatory, *n* (%)	0/113 (0.00)	0/95 (0.00)	-

*p*-Values were obtained with Mann–Whitney U tests and Pearson’s χ^2^ tests, respectively. Data are expressed as median (25th–75th percentile range) or a percentage of N. ^‡^ Participants with levels above the detection limit. Abbreviations: HIV, human immunodeficiency virus; ART, antiretroviral therapy; NT-proBNP, N-terminal pro b-type natriuretic peptide; ADAMTS13, a disintegrin and metalloproteinase with a thrombospondin type 1 motif, member 13; eGFR, estimated glomerular filtration rate.

**Table 2:** Independent associations between cardiovascular measures and viremia status (*n* = 205).

	Central SBP	PWV	PPA	IMT	Distensibility	Troponin T	NT-proBNP
	*β* (95%CI)	*β* (95%CI)	*β* (95%CI)	*β* (95%CI)	*β* (95%CI)	*β* (95%CI)	*β* (95%CI)
**Univariate**							
Low-level viremia ^‡^	−0.02 (−0.31;0.24)	−0.03 (−0.35;0.23)	−0.06 (−0.39;0.15)	−0.05 (−0.40;0.19)	−0.08 (−0.49;0.16)	−0.08 (−0.46;0.14)	−0.11 (−0.50;0.06)
**Multivariate**							
Low-level viremia ^‡^	0.01 (−0.22;0.26)	−0.04 (−0.31;0.14)	−0.07 (−0.36;0.08)	−0.02 (−0.29;0.23)	−0.11 (−0.52;0.07)	−0.05 (−0.38;0.19)	−0.13 (−0.52;0.02)
Sex, men	−0.14 (−0.61;−0.03) *	0.11 (−0.03;0.52)	0.16 (0.01;0.68) *	0.12 (−0.04;0.60)	−0.04 (−0.44;0.27)	0.21 (0.15;0.86) *	−0.19 (−0.75;−0.09) *
Age	0.33 (0.16;0.49) **	0.32 (0.15;0.47) **	−0.31 (−0.45;−0.13) **	0.43 (0.26;0.63) **	−0.37 (−0.58;−0.16) *	0.06 (−0.15;0.26)	0.23 (0.03;0.42) *
WC	0.23 (0.09;0.35) *	−0.20 (−0.32;−0.07) *	0.07 (−0.05;0.19)	0.12 (−0.03;0.26)	−0.13 (−0.29;0.04)	0.12 (−0.04;0.28)	−0.10 (−0.24;0.06)
Cholesterol	0.07 (−0.07;0.21)	0.03 (−0.10;0.15)	−0.01 (−0.13;0.11)	−0.07 (−0.23;0.07)	−0.07 (−0.24;0.10)	−0.04 (−0.21;0.12)	−0.15 (−0.30;0.002)
Glucose	0.03 (−0.09;0.15)	0.07 (−0.05;0.18)	0.05 (−0.07;0.15)	0.01 (−0.12;0.14)	−0.06 (−0.21;0.09)	0.10 (−0.05;0.24)	−0.07 (−0.20;0.08)
GGT	0.15 (0.02;0.28) *	−0.02 (−0.14;0.11)	−0.14 (−0.26;−0.01) *	0.06 (−0.08;0.21)	−0.22 (−0.39;−0.06) *	0.01 (−0.15;0.17)	0.11 (−0.04;0.26)
Smoke, yes	0.04 (−0.17;0.34)	0.08 (−0.08;0.39)	0.10 (−0.04;0.42)	−0.01 (−0.29;0.27)	0.02 (−0.28;0.35)	0.10 (−0.10;0.51)	0.03 (−0.23;0.34)
eGFR	0.05 (−0.11;0.21)	0.11 (−0.04;0.25)	−0.07 (−0.21;0.08)	−0.04 (−0.22;0.13)	−0.14 (−0.34;0.05)	−0.01 (−0.20;0.18)	−0.13 (−0.31;0.05)
*MAP*	n/a	0.53 (0.40;0.66) **	−0.17 (−0.29;−0.04) *	0.02 (−0.13;0.17)	−0.001 (−0.17;0.17)	0.19 (0.03;0.37) *	0.14 (−0.01;0.30)
*Heart rate*	n/a	n/a	0.47 (0.34;0.57) **	n/a	n/a	n/a	n/a
*Height*	n/a	n/a	0.16 (0.02;0.29) *	n/a	n/a	n/a	n/a
Adjusted *R^2^* of model	0.26	0.43	0.37	0.24	0.21	0.11	0.11

*p*-values were obtained with multivariate linear regression analyses (enter method). * *p* ≤ 0.050; ** *p* ≤ 0.001. ^‡^ Where 0 = suppressed viremia and 1 = low-level viremia. All multivariate models were significant and VIF values were ≤224 for all variables. NS: did not enter the final model. Abbreviations: n/a, not applicable (not part of model); SBP, systolic blood pressure; PWV, pulse wave velocity; PPA, pulse pressure amplification; IMT, intima-media thickness; NT-proBNP, N-terminal pro b-type natriuretic peptide; WC, waist circumference; GGT, gamma-glutamyl transferase; eGFR, estimated glomerular filtration rate; MAP, brachial mean arterial pressure.

**Table 3:** Odds of having cardiovascular measures and biochemical markers above the 75th percentile while having low-level viremia (*n* = 205).

	Odds Ratio (95% CI)	*p*-Value
**Cardiovascular measures**		
Central systolic blood pressure (≥130 mmHg)	1.19 (0.57;2.46)	0.65
Pulse wave velocity (≥8.6 m/s)	2.40 (0.90;6.41)	0.08
Pulse pressure amplification (≥1.43)	1.03 (0.44;2.44)	0.94
Carotid intima-media thickness (≥0.65 mm)	0.72 (0.32;1.63)	0.43
Carotid diameter distensibility (≥12.3%)	1.09 (0.50;2.39)	0.82
Troponin-T (≥3.01 pg/mL)	1.31 (0.58;2.97)	0.52
NT-proBNP (≥104 pg/mL)	1.42 (0.68;2.99)	0.35
**Other biomarkers**		
C-reactive protein (≥7.15 mg/L)	0.68 (0.32;1.44)	0.32
Interleukin-6 (≥4.42 pg/mL)	0.70 (0.35;1.39)	0.31
Intercellular adhesion molecule-1 (≥47.8 ng/mL)	1.24 (0.62;2.45)	0.54
Vascular cell adhesion molecule-1 (≥627 ng/mL)	0.79 (0.40;1.53)	0.48
P-selectin (≥36.6 ng/mL)	0.71 (0.37;1.39)	0.32
Myeloperoxidase (≥94.4 ng/mL)	1.63 (0.83;3.21)	0.16
Growth differentiation factor-15 (≥0.92 ng/mL)	1.60 (0.73;3.49)	0.24
ADAMTS13 (≥474 ng/mL)	1.07 (0.53;2.18)	0.85
Reactive oxygen species (≥247 Units)	0.67 (0.34;1.28)	0.22

Standardized odds ratios, 95% confidence intervals and *p*-values were obtained with binomial logistic regression analyses (enter method). 0 = suppressed viremia and 1 = low-level viremia. Abbreviations: NT-proBNP, N-terminal pro b-type natriuretic peptide; ADAMTS13, a disintegrin and metalloproteinase with a thrombospondin type 1 motif, member 13. Variables entered into the models included viremia status, sex, age, waist circumference, cholesterol, glucose, gamma-glutamyl transferase, tobacco use, estimated glomerular filtration rate, and additionally mean arterial pressure (for Troponin T, NT-proBNP, pulse wave velocity, intima-media thickness, distensibility and all biomarkers) or mean arterial pressure, heart rate and height (for pulse pressure amplification).

## Data Availability

The data presented in this study are available on request from the corresponding author.

## Supplementary Materials

The following supporting information can be downloaded at: https://www.mdpi.com/article/10.3390/jcm11102812/s1, Table S1: Difference in cardiovascular measures and biomarker levels between people with low-level viremia (LLV) and suppressed viremia, stratified by sex.
