# Supplementary material for: Cardiovascular Profile of South African Adults with Low-Level Viremia during Antiretroviral Therapy
Source: J Clin Med. 2022 May 16;11(10):2812. doi: 10.3390/jcm11102812 (PMC9144153; doi:10.3390/jcm11102812)
Supplement: Supplementary file 1 [file jcm-11-02812-s001.zip › jcm-1711295-supplementary.pdf]

Supplementary Table S1. Difference in cardiovascular measures and biomarker levels between people with low-level viremia (LLV) and suppressed viremia, stratified by sex.

	Women				Men			
	**SV** *n*=81	**LLV** *n*=72	***P*-value**	**B-H**	**SV** *n*=32	**LLV** *n*=23	***P*-value**	**B-H**
Age (years)	44.0 (34.5;51.0)	43.0 (35.3;49.0)	0.98	0.98	48.0 (43.0;53.0)	44.0 (37.0;48.0)	0.073	0.73
Body mass index (kg/m2)	26.9 (22.9;32.0)	26.3 (22.8;32.3)	0.84	0.98	20.4 (17.9;22.8)	20.3 (18.2;23.7)	0.99	0.99
**Cardiovascular measures**								
Brachial SBP (mmHg)	114 (106;131)	114 (103;131)	0.74	0.98	123 (106;130)	113 (108;121)	0.13	0.73
Brachial DBP (mmHg)	82 (75;88)	83 (74;94)	0.32	0.98	80 (73;91)	80 (74;88)	0.89	0.92
Brachial MAP (mmHg)	92 (85;104)	93 (83;106)	0.60	0.98	93 (86;105)	93 (85;98)	0.51	0.73
Central SBP (mmHg)	114 (108;131)	114 (107;130)	0.93	0.98	113 (103;132)	111 (99;118)	0.21	0.73
Central pulse pressure (mmHg)	35 (30;41)	34 (29;42)	0.53	0.98	34 (26;37)	29 (27;33)	0.14	0.73
Heart rate (beats/min)	69 (62;79)	72 (65;79)	0.40	0.98	69 (60;83)	66 (60;89)	0.77	0.88
Pulse wave velocity (m/s)	7.30 (6.40;8.30)	7.30 (6.40;8.30)	0.87	0.98	8.30 (7.53;9.08)	8.10 (7.40;8.50)	0.19	0.73
Pulse pressure amplification	1.32 (1.25;1.41)	1.32 (1.26;1.41)	0.90	0.98	1.42 (1.32;1.50)	1.39 (1.32;1.52)	0.75	0.88
Carotid intima-media thickness (mm)	0.60 (0.56;0.64)	0.59 (0.57;0.64)	0.47	0.98	0.63 (0.59;0.69)	0.61 (0.57;0.69)	0.46	0.73
Carotid diameter distensibility (%)	9.93 (7.16;12.8)	8.47 (6.53;12.4)	0.16	0.94	8.21 (5.07;11.9)	9.11 (4.63;12.0)	0.89	0.92
Troponin-T, >3.00 (pg/ml)*	5.04 (3.78;5.88)	4.53 (3.46;5.84)	0.78	0.98	4.75 (3.62;11.0)	5.82 (4.12;11.5)	0.77	0.88
NT-proBNP (pg/ml)	52.1 (22.8;106.0)	46.8 (23.2;97.3)	0.84	0.98	51.3 (20.2;144.9)	21.5 (7.59;40.6)	**0.007**	0.23
**Other biomarkers**								
C-reactive protein (mg/l)	2.44 (1.27;7.51)	3.82 (0.97;8.84)	0.66	0.98	2.57 (0.91;7.76)	0.98 (0.40;4.43)	0.082	0.73
Interleukin-6 (pg/ml)	2.44 (1.54;3.79)	2.83 (2.06;4.94)	**0.034**	0.56	3.11 (1.70;4.82)	2.01 (0.75;3.21)	0.19	0.73
ICAM-1 (ng/ml)	26.6 (17.6;45.1)	26.6 (21.2;42.3)	0.57	0.98	41.0 (24.2;62.4)	38.7 (29.5;46.4)	0.75	0.88
VCAM-1 (ng/ml)	493 (363;625)	460 (367;609)	0.72	0.98	519 (388;600)	557 (476;740)	0.19	0.73
P-selectin (ng/ml)	26.9 (21.5;34.7)	26.3 (19.5;35.0)	0.74	0.98	31.5 (21.5;40.9)	33.9 (28.8;44.3)	0.23	0.73
Myeloperoxidase (ng/ml)	66.8 (44.3;97.2)	55.9 (35.2;89.3)	0.061	0.67	70.3 (38.4;101.3)	85.9 (45.1;108.7)	0.49	0.73
GDF-15 (ng/ml)	0.55 (0.40;0.90)	0.63 (0.44;0.76)	0.63	0.98	0.86 (0.52;1.42)	0.78 (0.66;1.16)	0.49	0.73
ADAMTS13 (ng/ml)	401 (340;469)	398 (362;458)	0.78	0.98	397 (296;512)	431 (342;499)	0.55	0.76
Reactive oxygen species (Units)	205 (159;242)	218 (175;250)	0.12	0.94	205 (167;264)	205 (163;301)	0.58	0.77
Cholesterol (mmol/l)	2.90 (2.39;3.76)	2.93 (2.56;3.35)	0.89	0.98	2.65 (2.19;3.28)	2.98 (2.47;3.48)	0.30	0.73
Triglycerides (mmol/l)	0.74 (0.56;1.09)	0.70 (0.49;1.07)	0.28	0.98	0.77 (0.53;1.22)	0.82 (0.55;1.46)	0.40	0.73
Low-density lipoproteins (mmol/l)	1.80 (1.26;2.38)	1.72 (1.45;2.06)	0.74	0.98	1.59 (1.15;2.11)	1.65 (1.30;2.23)	0.44	0.73

High-density lipoproteins (mmol/l)	1.03 (0.82;1.29)	1.06 (0.81;1.36)	0.91	0.98	1.09 (0.77;1.34)	0.98 (0.67;1.21)	0.28	0.73
Apolipoprotein A (g/l)	1.16 (0.97;1.47)	1.16 (1.00;1.38)	0.95	0.98	1.17 (0.92;1.46)	1.12 (0.88;1.48)	0.49	0.73
Apolipoprotein B (g/l)	0.60 (0.45;0.78)	0.60 (0.49;0.74)	0.66	0.98	0.59 (0.43;0.68)	0.58 (0.46;0.79)	0.42	0.73
Glycated hemoglobin (%)	5.51 (5.19;5.76)	5.35 (5.18;5.66)	0.17	0.94	5.30 (5.07;5.62)	5.45 (5.17;5.66)	0.28	0.73
Glucose (mmol/l)	3.72 (3.31;4.38)	3.40 (3.16;3.79)	**0.004**	0.13	3.43 (3.15;3.76)	3.64 (3.23;4.22)	0.46	0.73
Gamma-glutamyl transferase (U/l)	37.6 (24.2;78.0)	33.7 (19.3;83.8)	0.64	0.98	64.9 (38.1;126.5)	60.6 (23.8;203.3)	0.87	0.92
eGFR (ml/min/1.73m^2)	121 (108;131)	120 (109;131)	0.72	0.98	115 (107;128)	120 (106;127)	0.40	0.73

P-values were obtained with Mann-Whitney U tests and Pearson's χ^2 tests, respectively. Data expressed as median (25th - 75th percentile range) or a percentage of N. *Participants with levels above the detection limit (Troponin-T: *n*=51, viral load: *n*=166). Abbreviations: SV, suppressed viremia; B-H, Benjamini-Hochberg *p*-value, SBP, systolic blood pressure; DBP, diastolic blood pressure; MAP, mean arterial pressure; NT-proBNP, N-terminal pro b-type natriuretic peptide; ICAM-1, intercellular adhesion molecule-1; VCAM-1, vascular cell adhesion molecule-1; GDF-15, growth differentiation factor-15; ADAMTS13, a disintegrin and metalloproteinase with a thrombospondin type 1 motif, member 13; eGFR, estimated glomerular filtration rate.
